# Survey of Extent of Translation of Oral Healthcare Guidelines for ICU Patients into Clinical Practice by Nursing Staff

**DOI:** 10.1155/2017/1348372

**Published:** 2017-10-18

**Authors:** Vivek Agarwal, Rameshwari Singhal, Richa Khanna, Pavitra Rastogi, Avinash Agarwal, Shuchi Tripathi

**Affiliations:** ^1^Department of Medicine, Mayo Institute of Medical Sciences, Barabanki, India; ^2^Department of Periodontology, Faculty of Dental Sciences, King George's Medical University, Lucknow, India; ^3^Department of Pedodontics, Faculty of Dental Sciences, King George's Medical University, Lucknow, India; ^4^Department of Medicine, Faculty of Medical Sciences, King George's Medical University, Lucknow, India; ^5^Department of Prosthodontics, Faculty of Dental Sciences, King George's Medical University, Lucknow, India

## Abstract

Nosocomial infections in critically ill/ventilated patients result from bacterial load in oropharyngeal regions. Oral decontamination serves as the easiest effective means of controlling infections. Knowledge, attitude, and practices followed by healthcare personnel in intensive care settings need to be assessed to implement concrete measures in health-care. Survey questionnaire was constructed and implemented following its validation on seventy nursing and paramedical staff working in government and private intensive care units throughout Lucknow city. 21-item questionnaire consisted of three parts of seven questions each. 78% of respondents had knowledge regarding oral care and its importance in critical settings but 44% of respondents considered it to be unpleasant task. 36% of respondents claimed to have provided oral care to all patients in ICU. Uniform guidelines for translation of oral healthcare in ICU settings are not being implemented. Previous studies in literature from various geographic diverse regions also point out to similar lacunae. Based on present survey, most respondents were aware of importance of oral care with protocols covered in academic curriculum. Attitude towards oral care is positive but respondents feel a need for specialised training. Practice for oral care is not sufficient and needs improvement and proper implementation.

## 1. Introduction

Hospital acquired infections (HAI) and nosocomial pneumonia results from colonisation of bacteria in the oral habitat of intensive care unit (ICU) patients. Microaspiration of oral and pharyngeal secretions is a major risk factor for nosocomial infections [1–4]. High mortality (>50%) with incidence rate of 9% to 27% occurs due to ventilator associated pneumonia (VAP) [5–8].

The two main interventions for decreasing bacterial load are selective decontamination of the digestive tract with administration of either oral antibiotics or through nasogastric tube and secondly oral decontamination through topical application of antiseptics or antibiotics.

Meta-analyses of decontamination of the digestive tract were found to be successful in reduction of ventilator associated pneumonia [9, 10]. Its use is limited due to the emergence of antibiotic resistant bacteria. Oral decontamination requires only a fraction of the antibiotics used in selective decontamination of the digestive tract and may be more useful in containing infection. Alternative to oral antibiotics is use of antiseptics, that is, chlorhexidine gluconate or povidone iodine. Antiseptics act rapidly at multiple target sites and are less prone to induce drug resistance.

Guidelines from the Center for Disease Control (CDC) recommend topical oral chlorhexidine 0.12% during the perioperative period for adults undergoing cardiac surgery [7]. Routine use of antibiotic/antiseptic oral decontamination for prevention of ventilator associated pneumonia is not yet subject to guidelines [7].

Serious nosocomial infections like VAP may be avoided by reducing pathogen colonisation of the oropharynx. Evidence based oral care protocols and preventive measures for VAP have been published. Little information exists on current practice, oral care training, and nurses' attitudes regarding the same. The primary purpose of this study was to identify the gap between what is known and what is done for oral care in Intensive care units. Knowing the differences between recommended and reported practices will allow for development, implementation, and evaluation of strategies that will have the potential to improve care outcomes. The objective of this survey was to assess the knowledge, attitude, and prevailing practises of paramedical and nursing staff involved in ICU regarding oral care for prevention of nosocomial infection.

## 2. Methods

### 2.1. Design

It is a cross-sectional survey of paramedical and medical staff working in various ICU's in government and private setups in Lucknow.

### 2.2. Respondents

The population of interest was critical care nurses and paramedical staff providing care for adult patients receiving mechanical ventilation in Lucknow city in an acute care setting.

### 2.3. Instrument

The Oral Care Questionnaire is an investigator-designed instrument to gather information from critical care nurses and paramedical staff on their knowledge, attitude towards oral care, and current care practices for adult patients in acute care setting.

The 21-item questionnaire was designed to gather information related to current oral care practices, training, and attitudes among paramedical and medical staff working in ICU regarding oral care.

The questionnaire was developed by the research team because of a lack of a previously developed and tested instrument and was based on the research questions and a review of the literature.

The research questions were as follows:

1. How are ICU health care workers (HCWs) trained in oral care?
2. What are the attitudes and beliefs of ICU HCWs regarding oral care?
3. What is the type and frequency of oral care provided to ICU patients?

A 5-point Likert scale of strongly agree, somewhat agree, neither agree nor disagree, somewhat disagree, or strongly disagree was used to assess respondents' attitudes and beliefs about oral care.

The survey includes questions about the oral care guidelines, presence of oral care protocols, use of oral chlorhexidine gluconate rinse, and questions to provide information about current oral care practices.

The questionnaire was translated in Hindi for ease of understanding of the paramedical staff.

### 2.4. Questionnaire Validation

Content validation of the survey was obtained by using a panel of 3 persons: an infection control nurse, a periodontist with knowledge about oral infection control, and a critical care expert physician. Each person was familiar with the oral care guidelines, and each received a copy of summary of the research published regarding oral care guidelines. Each commented on the adequacy of the match between the guidelines and the questions on the survey. No additional items were suggested, and no items were suggested for deletion or revision.

The survey was then distributed to 10 paramedical and medical staff workers in ICU from KGMU and one private hospital employed in a variety of ICU settings to evaluate readability of questionnaire and time to complete the survey. None of these 10 respondents had questions or concerns about the questions, and they were able to complete the survey within 5 minutes. Three of these nurses completed the survey again 1 week later, and their responses were highly similar to those from the first time they completed the survey.

### 2.5. Survey

The survey was distributed to 70 paramedical and medical staff from various hospitals employed in a variety of ICU settings.

#### 2.5.1. Blinding

A cover sheet on each survey instructed respondents to protect anonymity by placing no identifying information like name, address, and designation on the survey.

#### 2.5.2. Consent

Respondents also were informed that completion and return of the surveys implied their consent to participate.

#### 2.5.3. Instrument Distribution and Collection

The permission to carry out the survey was taken from the ICU in charge of the unit concerned. The survey questionnaire was given to the respondents and they were asked to complete the form at that time only. Therefore, we were able to assess the spontaneous response rather than manipulated response if the survey questionnaire was left with the respondents to return at their leisure.

#### 2.5.4. Data Collection and Analysis

The collected data was compiled electronically. The Likert scale analysis was done and percentage calculated for each response. Thus, each item was analysed separately. Further, a summative analysis was done on three parameters:

1. Knowledge
2. Attitude
3. Practise.

## 3. Results

### 3.1. Description of the Sample

A total of 70 surveys were distributed. 68 respondents completed the survey. 16 surveys were discarded because they were less than 30% completed. Further two were randomly discarded to make the sample size of 50 respondents.

### 3.2. Knowledge Assessment

The results suggest that maximum number (78%) strongly agrees that oral care is important in seriously ill patients admitted in ICU. The education regarding oral care is not adequate for ICU patients. There is mixed feeling among respondents regarding importance of oral care only for ventilator ridden patients (20 agree; 20 disagree; and 10 somewhat agree).

Most respondents agree with clear cut oral care outlined manuals. Most agree with the use of electronic brushes over manual brushes. However most disagree with clear guidelines, concentration, and frequency of antiseptic use. Most respondents also disagree about being periodically updated regarding recent advances in oral care in ICU (Table 1).

### 3.3. Attitude Assessment

Most respondents believe there is a need for training ICU staff for oral care especially specialised training. There is agreement regarding the training only for workers in ICU setups but the agreement is not strong. There is no consensus regarding the oral care being provided only after doctor's insistence (50% agree and 40% disagree). Most agree that dental hygienists may be employed for carrying out oral care; however most disagree with doctors providing oral care themselves. Majority of respondents believe providing oral care to be an unpleasant job (Table 2).

### 3.4. Practise Assessment

Regarding oral care practises, most respondents agree with oral care provided to all seriously ill patients in ICU and majority somewhat agree with it being provided to only ventilator associated patients. Majority agree with the use of chlorhexidine gluconate 0.12% at a frequency of twice daily to be used for oral care practise in ICU. Respondents agree with the reduction of nosocomial infections in patients receiving adequate oral care. However, electronic brushes are not used frequently. Majority of respondents believe in having adequate knowledge regarding oral care in ICU patients but the practise of the same is not adequate (Table 3).

## 4. Discussion

The present survey describes a questionnaire based assessment of the knowledge, attitude, and practise among healthcare workers in a critical setup for increased prevalence of nosocomial infections.

Little information is available in the literature regarding oral care practices, and only one validated instruments could be found to address the research questions presented in our study [11]. Binkley et al. survey was conducted in US ICU setting. The present survey was designed to assess work practises, nursing training, and practises in Indian scenario.

Nursing programs today ensure that nursing students master a wide variety of complex and technology-driven skills that supersede the simple task of oral care. This survey was designed to assess the current knowledge and practises in ICU nursing and paramedical staff. The information gathered herein may be utilised for formulating protocols for enhanced training and practise of oral care in ICU setups.

According to the nurses' self-reports from various studies, evidence based best practises for the prevention of nosocomial infections are not consistent and uniform in their implementation [11, 12].

In the present study, the knowledge assessment showed that the respondents are aware of the importance of oral care in seriously ill patients. They are well taught about oral care and have clearly defined manuals. They are also aware about electronic brushes being better in proving oral care than manual brushes. The antiseptic concentration and frequency of use is a point of confusion among most of the respondents (somewhat agree 42%). The lacuna in their education regarding oral care protocols is lack of knowledge regarding recent advances and participation in continuing education programs (Table 1).

The efficacy of the electric toothbrush for dental plaque removal and promotion of gingival health has been well proven [13]. A review of the certified nursing curriculum indicates that oral care is a focused skill. The activities involved in the process are not well defined and are guided by the individual procedures protocol of the employing organization. Although the basic skills are reinforced in a professional setting and rationale for actions emphasized but they still need to be incorporated in the certified nursing program where initial impressions are made.

This survey shows that nurses have a positive attitude towards providing oral care practice to mechanically ventilated patients. The finding is similar to the survey by Binkley et al. [11]. In another study 40% to 46% of respondents reported that oral care is an unpleasant and difficult task, and the mouth of the patient who needs prolonged ventilation deteriorates even after oral care [14]. In our survey 78% of the nurses regarded oral care as very important for the seriously ill patients and had the training and time to provide it. These results are similar to those of a study by Binkley et al. [11]. Having sufficient time to provide oral care, prioritizing oral care and not finding it unpleasant are associated with better oral care practises [15]. Another survey on oral care interventions in the ICUs found that oral care was accorded low priority while greater importance was given to stabilising the condition of the critically ill patients [16].

Nurses are hesitant to provide oral care or use toothbrush for patients who are intubated as endotracheal tubes may limit access to the oral cavity [17]. Nurses also fear dislodging/displacing the endotracheal tube [18]. Nurses' hesitation in using toothbrushes is due to their lack of knowledge of recent research findings [19]. Therefore, nurses were not providing a variety of oral care interventions designed for patient's comfort rather than plaque removal [20].

The gap between what we know and the way we practice continues to be larger than desired. Nearly 70% of respondents agree with the same (54% strongly agree and 16% agree). Nurses' compliance with recommendations about oral care must be improved. Few nurses did not use toothbrushes in their practice but followed the oral care protocol learned during their nursing training. It shows that they have positive attitude in providing oral care. The nurses also practiced oral care as a routine procedure.

Chlorhexidine is an easily applied and relatively inexpensive preventive measure with minimal side effects. Tooth brushing at least twice per day has been shown to reduce pneumonia in dependent patients and is more cost-effective than routine use of foam swabs [21]. The present survey suggests that most respondents strongly agree with the use of chlorhexidine (64%) with a frequency of twice daily (44%).

Oral care is an important component of nursing and various protocols for oral care practises have been proposed [21–25]. Powered toothbrushes have been shown to improve the quality of care and are easier to use than manual brushes [26]. In the present study 68% of respondents did not use powered toothbrushes in their practice. The major reason could be the cost involved in procuring electric toothbrushes.

## 5. Conclusion

Based on the present survey, most respondents were aware of the importance of oral care with protocols covered in the academic curriculum. The attitude towards oral care is positive but the respondents feel a need for specialised training. The practice for oral care is not sufficient and needs improvement and proper implementation.

The limitations for the present study could be a small sample size and nondescriptive format. Further studies can be planned gaining knowledge regarding the oral care protocols followed in various ICUs and formulation of a standardised protocol.

## Figures and Tables

**Table 1:** Assessment of knowledge regarding oral care in ICU setting.

Questions	Strongly agree	Agree	Somewhat agree	Do not agree	Strongly disagree
(1) Oral care important for patients in ICU	78%	16%	6%	0%	0%
(2) Nursing students adequately trained in oral care	20%	16%	36%	20%	8%
(3) Oral care important only for ventilated patients	16%	24%	20%	14%	26%
(4) Protocols for oral care clearly outlined in ICU care manuals	24%	36%	4%	10%	26%
(5) Electronic brushes are better than conventional cleaning methods	34%	24%	22%	14%	6%
(6) Periodical updating on recent advances in oral care	4%	26%	18%	42%	10%
(7) Adequate knowledge about oral antiseptic use, its frequency and concentration	6%	18%	42%	22%	12%

**Table 2:** Assessment of attitude towards oral care in ICU setting.

Questions	Strongly agree	Agree	Somewhat agree	Do not agree	Strongly disagree
(1) Need to educate ICU staff regarding oral care	46%	24%	4%	18%	12%
(2) Specialized training in oral care in seriously ill patients needed	48%	32%	28%	18%	4%
(3) Required only for those care givers who work in ICU setups	6%	20%	28%	14%	12%
(4) Nursing staff follows oral care practises properly only if doctor insists	26%	24%	10%	22%	4%
(5) Need to involve dental hygienists to give better oral care in ICU settings	54%	16%	4%	22%	4%
(6) Doctors should preferably do oral care themselves	2%	18%	14%	38%	28%
(7) Providing oral care is an unpleasant task	26%	44%	14%	10%	2%

**Table 3:** Assessment of practises of oral care followed in ICU setting.

Questions	Strongly agree	Agree	Somewhat agree	Do not agree	Strongly disagree
(1) Oral care given to all patients in ICU	24%	36%	18%	14%	8%
(2) Oral care given only to artificially ventilated and comatose patients	6%	16%	58%	18%	2%
(3) Chlorhexidine 0.12% is best for oral care	64%	18%	2%	14%	2%
(4) Frequency of oral care is at least twice daily	44%	24%	18%	105	4%
(5) Nosocomial infections reduced in patients who receive adequate oral care	18%	42%	28%	12%	0%
(6) Electronic brushes used more frequently for daily oral care	0%	4%	26%	68%	8%
(7) Tooth pastes used along with brush for oral cleaning	54%	16%	4%	22%	4%

## Additional Points

*Recommendations*. (i) There is a need for specialised continuing education programs to update ICU staff regarding recent advances in oral care for prevention of HAI. (ii) There is need for developing standardised protocols for oral care in ICU related to duration, frequency, type of antiseptic, type of brush, and so on. (iii) A dental hygienist may be employed for carrying out oral care in ICU. (iv) Provision of electronic brushes can be made in ICUs.
